# Failure Behavior and Mechanism of Solder Joint Under Thermal Mechanical Coupling Loads

**DOI:** 10.3390/ma19030640

**Published:** 2026-02-06

**Authors:** Yuxin Deng, Si Chen, Peijiang Liu, Guoguang Lu, Xiaofeng Yang, Yu Zhao, Xiaodong Jian

**Affiliations:** 1 School of Material and Energy, Guangdong University of Technology, Guangzhou 510006, China; 2112302094@mail2.gdut.edu.cn; 2 The Science and Technology on Reliability Physics and Application of Electronic Componen Laboratory, The 5th Electronics Research Institute of the Ministry of Industry and Information Technology, Guangzhou 510610, China; chensiceprei@yeah.net (S.C.); cz2343222@163.com (P.L.); luguog@126.com (G.L.); yxf004@hotmail.com (X.Y.)

**Keywords:** solder joint, temperature cycling, thermal–mechanical coupling, fatigue life

## Abstract

The periodic thermal loads to which electronic devices are exposed during operation induce alternating thermal stresses due to the mismatched coefficients of thermal expansion (CTE) between the solder joints and the surrounding materials. This leads to cyclic thermal strain, ultimately causing crack initiation, propagation, and failure of interconnect structures. This study investigates thermal fatigue failure of Sn3.5Ag solder joints induced by cyclic thermal stresses from CTE mismatch. Numerical simulations and experiments reveal that alternating shear strain concentrates at the joint–pad interface, serving as the crack initiation site. This study proposes a hypothesis: extracting the equivalent viscoplastic strain range from the steady-state hysteretic response after cyclic stabilization and applying it to the Coffin–Manson model can mitigate the strain overestimation inherent to methods based on the initial transient impact, thereby providing a more reasonable physical basis for thermal fatigue life evaluation. Based on this, the thermal fatigue life of the solder joint is predicted to be 18,930 cycles. Analysis confirms significantly higher viscoplastic strain energy density at this critical point, indicating energy dissipation drives damage. This study addresses the above hypothesis from three aspects: deformation mechanism, cyclic response, and energy dissipation, providing a key basis for developing a highly reliable method for assessing solder joint life.

## 1. Introduction

With the deepening development of information technology, electronic devices have become an integral part of every industry. Their system reliability is highly dependent on the manufacturing quality and performance of electronic components [1,2,3,4]. In the field of integrated circuits, technological evolution is advancing from the “post-Moore era” toward the “Super-Moore era.” This evolution continuously drives the development of electronic systems toward miniaturization, high-speed operation, and functional integration [5,6,7,8]. This trend also presents significant challenges. The demand for high-density integration necessitates continuous miniaturization of solder joint dimensions, resulting in the coexistence of multi-scale features within packaging structures and amplified geometric scale differences [9]. Against this backdrop, solder joints emerge as the weakest link in the entire packaging structure due to their stress concentration effects and interface-dominated mechanisms. Research indicates that the mechanical properties of micro-solder joints exhibit significant size effects, in which the yield strength increases exponentially with decreasing scale, while ductility declines sharply. This shift in mechanical behavior directly lowers the crack initiation threshold, shortens fatigue life, and ultimately severely compromises the long-term reliability of electronic devices through cross-scale failure chain propagation [9,10,11,12,13].

Statistics from the U.S. Air Force Electronics Industry Department indicate that temperature cycling is the primary cause of electronic component failure, accounting for approximately 55% of all failure causes [14,15]. During actual service, electronic products endure thermal shock loads due to periodic power cycling of circuits, inducing alternating thermal stresses within components. Under thermal cycling, mismatched thermal expansion coefficients between packaging materials generate periodic stresses at solder joints. These stresses drive the initiation and propagation of fatigue cracks, ultimately leading to joint fracture and functional failure [16,17,18,19]. As electronic packaging continues to evolve toward miniaturization and higher density, the risk of solder joint failure intensifies further. On the one hand, the miniaturization of solder joints leads to a dramatic increase in current density, significantly accelerating the electromigration failure process. On the other hand, the increased integration of chips results in a continuous rise in heat flux density, making heat dissipation an increasingly prominent issue. The combined effect of these factors triggers strong nonlinear coupling across thermal, electrical, and mechanical multiphysics domains, resulting in a complex mechanism of solder joint failure [20,21,22,23,24,25]. Given the strongly nonlinear constitutive behavior exhibited by welded joint materials, traditional analytical methods struggle to accurately describe their response. Consequently, the adoption of multiscale modeling and simulation techniques has become a critical issue for achieving a precise understanding of failure mechanisms and reliable prediction of fatigue life [26,27,28].

The solder joint material belongs to a low-melting-point metal system, exhibiting multi-mechanism coupled deformation behavior under thermo-mechanical coupled loading. The mechanisms include elastic deformation, plastic slip, and diffusion-dominated creep [29,30,31]. Therefore, constructing a constitutive model capable of accurately describing its stress–strain response has become a core challenge in finite element analysis of interconnect solder joints. The key lies in quantifying the complex mechanical behavior under multiphysics coupling [32,33,34,35,36]. As electronic packaging continues to evolve toward high-density integration, the adoption of multiscale constitutive models to accurately characterize the mechanical response of solder joints has become a decisive factor in enhancing the precision of reliability simulations. Against this backdrop, Anand [37] proposed a unified constitutive model based on a single internal variable in the 1980s, initially designed to describe the inelastic deformation of metals during high-temperature forming processes. This model provided a theoretical framework for viscoplastic behavior and was significantly refined by Brown et al. [38] in 1989. With the widespread adoption of lead-free solder technology, the Anand model has been extensively applied to characterize the viscoplastic behavior of solder joints due to its theoretical completeness and applicability, becoming a vital tool for simulation studies in this field [39,40].

To achieve precise finite element prediction of solder joint fatigue life, researchers have developed various life prediction models. Su et al. [41] first systematically categorized and summarized four major types of mainstream models (plastic strain models, creep damage models, damage accumulation models, and energy models) to establish a clear classification framework. Building upon this foundation, Depiver [42] systematically evaluated the joint-life predictive capabilities of classical models, including Coffin–Manson, Engelmaier, Solomon, and Syed, subsequently developing a novel prediction model driven by a combination of damage parameters. To further enhance model accuracy and correlate with micro-damage mechanisms, Wang [43] employed a cross-scale correlation approach to establish a theoretical mapping relationship between equivalent cumulative plastic strain increments and wafer-level package thermal cycle life. This relationship was used to calibrate the parameters of the Coffin–Manson model, thereby constructing a unified mechanical–thermal fatigue life prediction framework. For more complex real-world operating conditions, Hu [44] employed multiphysics-coupled simulations to reveal the plastic strain-dominated fracture mechanism of lead-free solder joints under combined electrical–thermal–vibration loading. By modifying the Coffin–Manson model parameters, they developed a quantitative life prediction model for thermo-electrical coupled environments, providing critical theoretical support for the reliability design and evaluation of ball grid array (BGA) packages under complex multiphysics conditions. Lederer [45] adopted an experiment–simulation coupling method based on a 20 kHz ultrasonic frequency and systematically predicted the fatigue crack initiation life of Sn3.5Ag solder joint at room temperature to 175 °C by using the viscoplastic Anand model combined with improved Coffin–Manson and Goodman damage criteria. It is worth noting that in order to overcome the limitation of traditional finite element simulation calculation efficiency, Rajaguru [46] found another way and proposed a two-dimensional semi-analytical integrated damage model of Sn3.5Ag solder interconnection based on open data parameter calibration, which realized efficient damage assessment without numerical simulation.

In summary, the trend toward miniaturization and high-density packaging in electronic devices has increasingly highlighted reliability issues in solder joints under thermo-mechanical coupled loads. Although the Anand constitutive model and various fatigue life prediction methods provide a foundation for research, existing studies still face challenges in precisely quantifying cyclic deformation mechanisms and ensuring the physical plausibility of life predictions. On the one hand, there is a lack of systematic quantitative characterization of in-plane shear deformation and its energy dissipation processes within solder joints, which are dominated by CTE mismatch. On the other hand, life predictions often fail to strictly distinguish between initial transient and steady-state cyclic conditions, limiting model accuracy.

To this end, this paper establishes a simulation research framework integrating simulation analysis and experiment, aiming to systematically reveal the failure mechanisms of welded joints under thermal cycling loads and achieve precise life prediction. In this study, a finite element simulation was carried out based on Anand’s viscoplastic constitutive model. Then, the dangerous area of the solder joint structure was located according to simulation and experiment. Subsequently, by analyzing stress–strain hysteresis loops at these critical points, the damage evolution process was quantitatively characterized through the cyclic viscoplastic strain energy density dissipated and viscoplastic strain amplitude. The physical validity of employing parameters from the stable cycle phase was confirmed. Finally, the modified Coffin–Manson model was applied for life prediction and validated against results from COMSOL’s built-in fatigue module. This approach provides theoretical foundations and methodological support for reliability design and optimization in high-density electronic packaging.

## 2. Finite Element Simulation

### 2.1. Geometric Modeling

**Figure 1:**
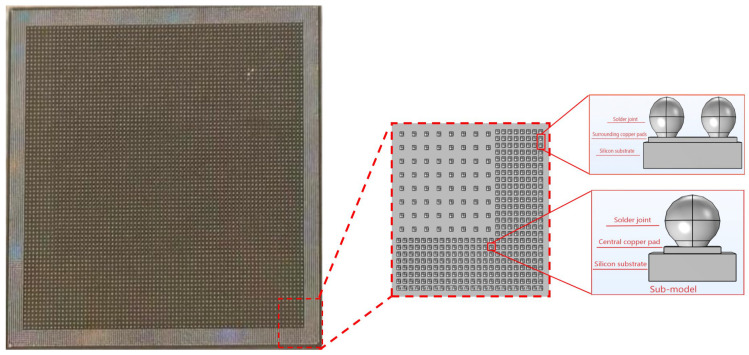
Contains a schematic diagram of the overall sample of the selected area.

Our study employs COMSOL Multiphysics 6.2 to establish a finite element model of micro-bump solder joints for thermal stress simulation. The model fully accounts for the structure’s symmetry and periodic characteristics. To enhance computational efficiency while maintaining accuracy, three simplification principles, including assumed uniform load distribution, zero defects in the structure, and homogenization of geometric dimensions across all material layers, are adopted. The calculation domain selects the 1/4 periodic symmetric region of the structure. At the same time, to analyze the sensitivity of the model’s material parameters, we selected a representative sub-region (see Figure 1) and constructed a sub-model. The boundary conditions of this sub-model were mapped from the physical field results of the global model. Figure 1 is a schematic diagram of the whole sample, including the selected area, and Table 1 lists the key geometric parameters.

### 2.2. Material Properties

Density, elastic modulus, Poisson’s ratio, thermal conductivity, and thermal expansion coefficient constitute the key input parameters for analyzing the stress–strain behavior of structural components under thermal cycling loads. Sn3.5Ag solder joints exhibit significant rate-dependent nonlinear mechanical behavior during thermal cycling, characterized by the Anand viscoplastic model. Model parameters are detailed in Table 2 [47,48,49], while material parameters for other structural components and Sn3.5Ag are listed in Table 3 and Table 4, respectively. Thus, to focus on the fatigue mechanism analysis of the solder joint structure under thermal cycling and to align with widely adopted simulation practices, this study employs well-established Anand parameters for Sn3.5Ag solder from Wang [48], which were derived through systematic testing and curve fitting. For other materials involved in the simulation, COMSOL built-in parameters were selected to ensure standardization and comparability. While the use of these recognized parameters provides a consistent and reliable simulation baseline, we acknowledge this as a limitation of the current work. Future studies will aim to improve accuracy by incorporating custom experimental measurements or more advanced constitutive models.

### 2.3. Thermal Simulation

**Figure 2:**
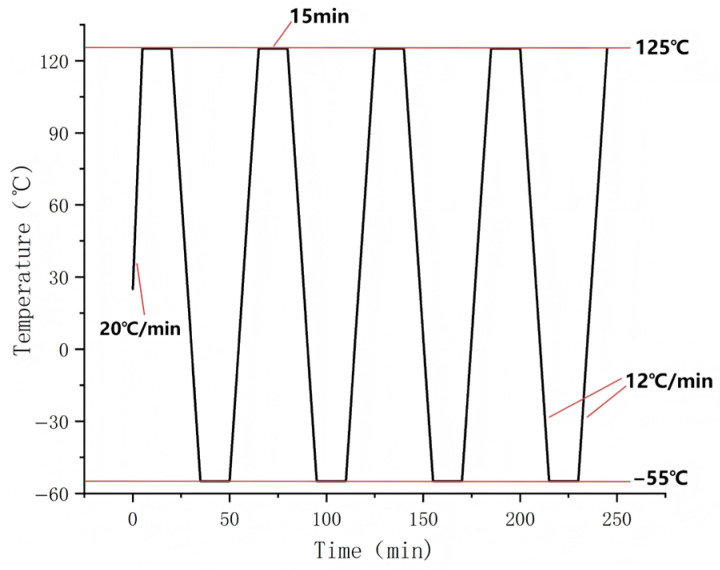
Temperature cycling load conditions.

**Figure 3:**
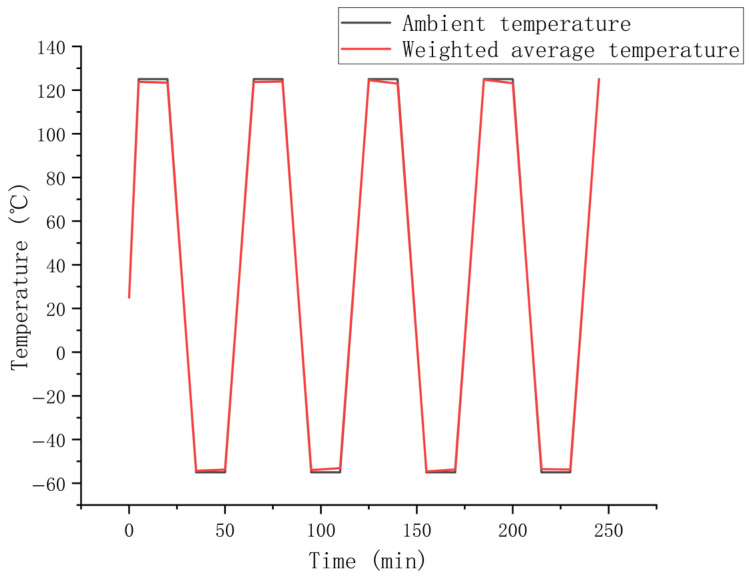
Ambient temperature and the weighted average temperature of the whole sample.

Our study establishes temperature cycling loads in accordance with the Joint Electron Device Engineering Council (JEDEC) standard JESD22-A104F.01 [50], with a temperature range of −55 °C to +125 °C. Thermal profiling begins by heating the sample from room temperature to the upper extreme at an initial rate of 20 °C/min. Subsequently, cyclic loading is applied within the specified temperature range, with each complete cycle lasting 60 min and maintaining a constant temperature change rate of 12 °C/min during both the heating and cooling ramps. The specific temperature cycling loading curve is shown in Figure 2. Both high and low temperature phases include a 15 min isothermal holding period, with an initial reference temperature set at room temperature (25 °C). In the simulation, it is assumed that the temperature distribution across the entire sample structure is instantaneously uniform. To validate this critical assumption, the volume-averaged temperature of the solder joint over time was extracted and is presented in Figure 3. A comparison reveals that this average joint temperature curve exhibits a high degree of coincidence with the applied ambient temperature profile, with a maximum recorded deviation of less than 3 °C. This close alignment quantitatively demonstrates that the effective temperature differential driving the thermal deformation is essentially consistent with the uniform temperature difference assumed in the model, thereby substantiating the validity of the simulation approach under the studied conditions.

The model employs a multi-physics coupled thermo-mechanical fatigue framework. The analysis is performed in two primary steps. First, the transient temperature distribution is calculated using the Heat Transfer in Solids physics interface. The resulting temperature field is then inherited as a thermal load for the subsequent transient structural analysis in the Solid Mechanics physics interface. Within the Solid Mechanics module, the following boundary conditions are applied: the bottom surface of the silicon substrate is prescribed as a fixed constraint, and the surfaces of the quarter-symmetric model region are defined with symmetry boundary conditions. To handle the highly non-linear nature of the problem, the solver employs a fully coupled approach using the implicit Backward Differentiation Formula (BDF) with a medium-accuracy time-stepping setting. Finally, the computed stress and equivalent viscoplastic strain fields are transferred to the fatigue module. The Coffin–Manson model is selected in this module, using the material’s fatigue toughness coefficient and fatigue toughness index as inputs to predict the fatigue life based on the equivalent viscoplastic strain amplitude obtained from the coupled thermo-mechanical simulation.

### 2.4. Experimental Methods

The samples to be tested were placed in a rapid temperature change test chamber (QW/T0570W/15T, Guangzhou-GWS Environmental Equipment Co., Ltd., Guangzhou, China) for thermal cycling tests. In this study, a total of 10 identical solder joint samples were cross-sectionally analyzed after interruption at different specified numbers of cycles—0 cycles (serving as the as-manufactured control group), 100 cycles, 200 cycles, and 300 cycles—to observe crack initiation using field emission scanning electron microscopy (SEM, Regulus8230, Hitachi, Tokyo, Japan). Since the packaging material itself is not conductive, conductive treatment was required before scanning electron microscope observation: first, sputtering gold onto the sample surface using a ion sputtering instrument (Q150TS, Quorum, Lewes, UK) to enhance conductivity; then, fixing the sample onto the sample stage with conductive adhesive to connect its upper and lower surfaces and establish a stable internal conductive path. Subsequently, a field emission scanning electron microscope equipped with an X-ray energy spectrometer was used to observe the cross-sections and fracture surfaces of the samples and to characterize element distribution. The SEM images presented in this paper were selected from samples in the corresponding cycle groups to illustrate typical morphological features.

## 3. Results and Discussion

### 3.1. Macroscopic Response Characteristics of Solder Joint Thermal Fatigue Behavior

**Figure 4:**
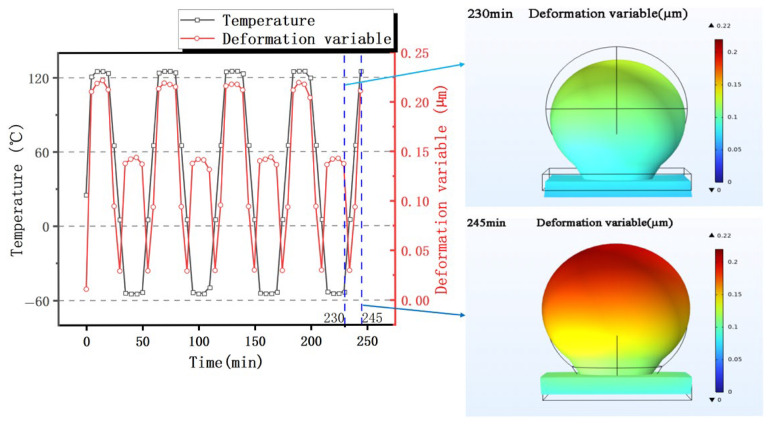
Deformation of solder joints at low and high temperatures.

As shown in Figure 4, the displacement morphology of the sub-model (refer to Figure 1 for location) under cyclic thermal loading is clearly discernible. During the low-temperature phase, the solder joint undergoes overall contraction deformation due to differences in the materials’ thermal expansion coefficients. Conversely, during the high-temperature holding phase, the solder joint exhibits pronounced bulging and protrusion morphology due to thermal expansion effects. This contraction and expansion deformation induced by temperature changes constitutes the core mechanism driving the periodic alternation of thermo-mechanical stresses within the solder joint, directly influencing its thermal fatigue life.

**Figure 5:**
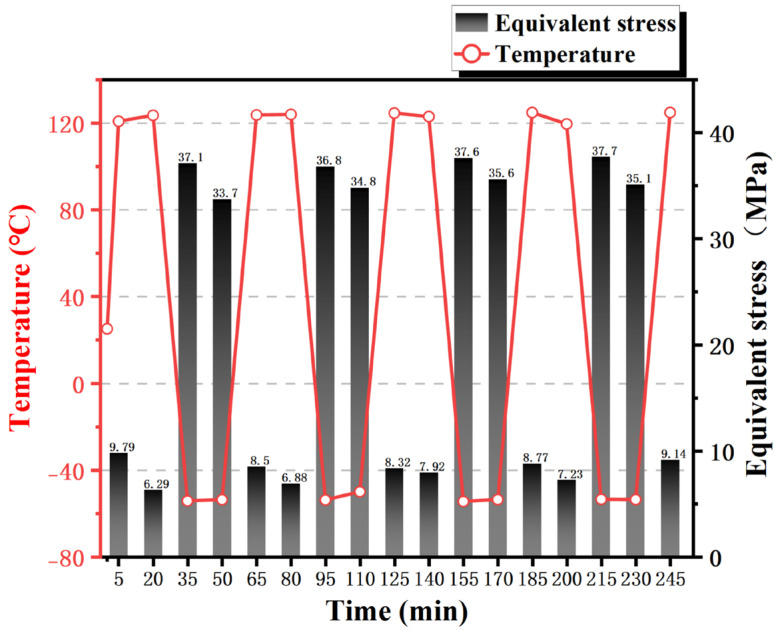
Maximum equivalent stress value map of the sample structure.

The maximum equivalent stress of the sample structure shown in Figure 5 fluctuates periodically with the temperature cycle. As the thermal expansion coefficient of the solder joint is larger than that of the silicon substrate, the solder joint mainly bears the compressive stress exerted by the solder joint at the initial stage of high temperature. Sn3.5Ag solder has a melting point of 220 °C. At elevated temperatures of 125 °C, enhanced atomic mobility leads to creep-dominated deformation, exhibiting pronounced viscoplastic behavior. During the high-temperature isothermal holding process, stress decreases over time under constant strain, forming stress relaxation. Consequently, the maximum equivalent stress slightly decreases upon completion of the high-temperature holding. The maximum stress value during the low-temperature initiation stage is significantly higher than that in the high-temperature stage. This can be attributed to three factors. Firstly, low temperatures suppress creep effects, reducing the capacity for plastic stress release and resulting in incomplete stress relaxation, which exacerbates stress concentration. Secondly, the elastic modulus of the solder joint increases with decreasing temperature, further amplifying thermal mismatch stresses. Finally, the synergistic effect of a high elastic modulus and low stress relaxation promotes crack initiation and propagation, becoming the primary mechanism for solder joint fatigue failure. During the low-temperature isothermal holding stage, the maximum stress value decreases slightly as residual stresses gradually relax. Notably, the initial heating rate of up to 20 °C/min induces thermal gradients within the material, leading to elevated equivalent stresses during the first five minutes. These stresses subsequently relax as the temperature distribution becomes more uniform.

The above macro-level analysis confirms that solder joints endure significant alternating thermo-mechanical stresses during thermal cycling. To elucidate the underlying micro-mechanisms and identify the precise failure locations, the subsequent discussion examines the localized evolution of equivalent viscoplastic strain, which serves as a key metric linking cyclic energy parameters to the initiation of fatigue damage. Although this physics-based framework is inherently universal, its quantitative generalizability across different material systems and loading conditions remains to be fully established and constitutes an important direction for future research.

### 3.2. Hazard Identification and Fatigue Damage Mechanism Analysis

**Figure 6:**
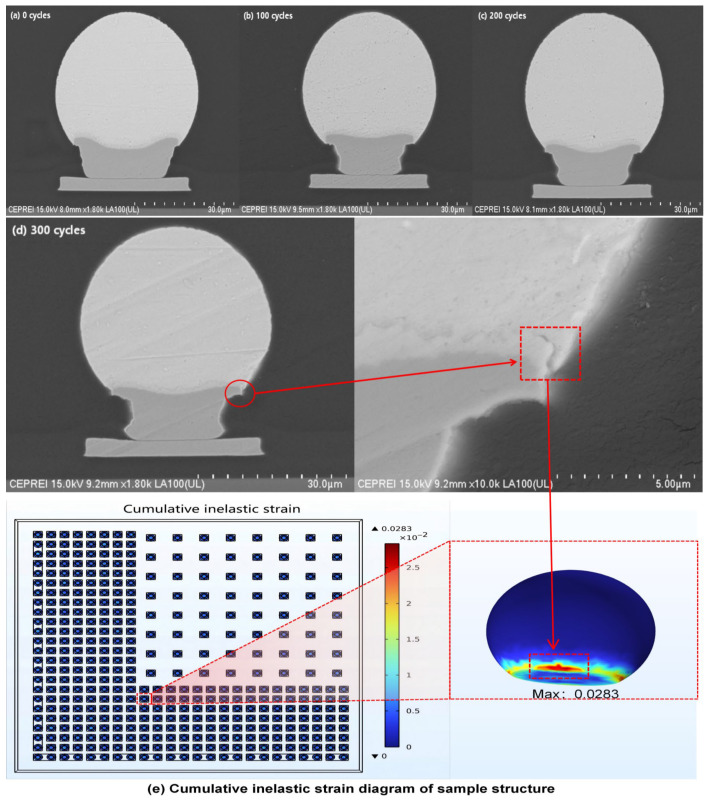
Correlation between experimental crack evolution and simulated strain accumulation under thermal cycling.

The fatigue failure of solder joints is fundamentally driven by the cyclic accumulation of inelastic strain (manifested predominantly as viscoplastic strain in solder joints), with the viscoplastic strain energy density dissipated per cycle serving as the core damage metric. The Anand constitutive model is employed to characterize this viscoplastic response and predict critical failure locations. Figure 6 integrally presents the correlation between simulation and experiment for this failure mechanism. The finite element analysis based on the Anand model (Figure 6e) identifies the weld interface edge between the solder joint and the pad as the location of maximum cumulative inelastic strain, marking it as the critical structural point. This spatial prediction is directly validated by experimental observation. The sequential SEM micrographs in Figure 6a–d illustrate the damage evolution, showing that the observable fatigue cracks initiated at the interface edge after 300 thermal cycles (Figure 6d) and propagated from this location. The precise agreement between the simulated strain concentration zone and the experimentally observed crack initiation site confirms that thermal fatigue failure is driven by strain concentration at the interface edge.

To elucidate the cyclic deformation mechanism underlying preferential failure at the edge regions of solder joints, deformation mechanisms and damage evolution processes are further analyzed. Deformation in this region primarily stems from the thermal expansion coefficient mismatch between the solder joint and the bonded pads in the XY plane, inducing cyclic plastic deformation dominated by shear deformation in the XZ and YZ planes within the solder joint [29,51,52]. The inelastic strain concentrated at the edges of the solder joint interface essentially reflects the plastic accumulation behavior driven by out-of-plane shear, which explains the phenomenon of fatigue crack initiation at this location from the perspective of deformation patterns.

**Figure 7:**
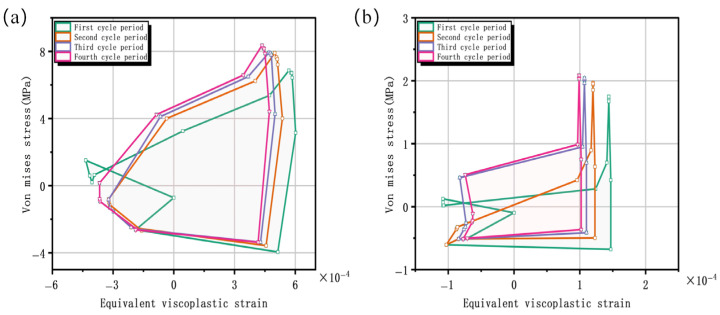
(**a**) Stress–strain hysteresis loop at the hazardous point. (**b**) Stress–strain hysteresis loop at the interface center point.

Figure 7 compares the stress–strain hysteresis loops at the critical point and at the interface center of the solder joint. The results indicate that the solder joint edge undergoes more significant cyclic plastic deformation and energy dissipation. This energy dissipation is the fundamental driver of fatigue damage accumulation. In order to further quantify the fatigue damage of solder joints, based on the energy method, the amplitude of viscoplastic strain energy density dissipated in each steady cycle is recorded as ΔWvp as the key damage parameter. This parameter is numerically equal to the area of the steady-state stress–strain hysteresis loop calculated by Anand’s viscoplastic constitutive model. Results indicate that ΔWvp reaches 0.00599 MJ/m^3^ at the critical point, which is approximately 30 times that (0.000203 MJ/m^3^) at the interface center point. This magnitude difference indicates that the critical point converts more mechanical work into irreversible damage energy during each thermal cycle, providing a sustained driving force for microcrack nucleation and propagation. It is confirmed from an energy perspective that this region sustains more significant irreversible damage with each cycle. This conclusion is completely consistent with the crack initiation position shown in Figure 6e, which is located at the edge of the interface between the solder joint and solder pad, and the two confirm each other. However, an important limitation must be noted: due to experimental constraints, we could not conduct the repeated life-to-failure tests needed to statistically prove that the highest ΔWvp region invariably fails first. Therefore, this statistical validation is designated as a critical objective for future work.

Comparing the hysteresis loop evolution at the critical point over the first four cycles reveals a trend toward stabilization in both shape and area after the initial two cycles. This study follows the established practice for energy-based fatigue analysis [53,54], adopting a relative change in the viscoplastic strain energy density of less than 5% between consecutive cycles as the engineering criterion for reaching a steady state. As shown in Table 5, the relative changes in the dissipated viscoplastic strain energy density between consecutive cycles from Cycle 2 to Cycle 4 are both 0.34%. These identical and minimal values are well below the 5% threshold. This confirms that the system reaches a steady state by the third cycle, indicating that the material response has entered a stable state with subsequent damage accumulating within this stabilized pattern. Therefore, the fourth cycle’s response is robust, and fatigue life predictions derived from these stabilized parameters are more physically justified than those relying on the initial transient response.

**Figure 8:**
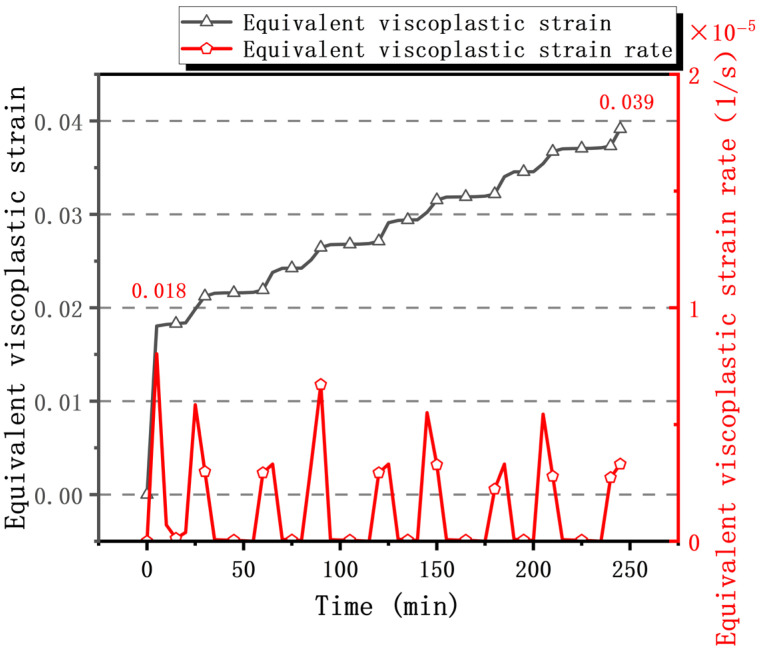
Equivalent viscoplastic strain and equivalent viscoplastic strain rate diagram for hazardous solder joints.

Figure 8 illustrates the evolution of equivalent viscoplastic strain and strain rate over time for the critical solder joint. It is evident that equivalent viscoplastic strain increases monotonically with time and cycle count, primarily due to high-temperature creep induced by alternating stresses during thermal cycling, failing to fully recover during the low-temperature phase. Although the strain increment per cycle is small, the cumulative effect of multiple cycles ultimately leads to fatigue failure or crack initiation in the solder joint. The maximum cumulative equivalent creep strain during the initial heating phase of the first cycle was 0.018. After three cycles, the total equivalent viscoplastic strain reached 0.039, with an average accumulation of 0.005 per cycle. Analysis of the equivalent viscoplastic strain rate further revealed its spatiotemporal distribution characteristics. During heating and cooling phases, the strain rate significantly increased, driving rapid strain growth. During the high-temperature holding stage, enhanced atomic mobility and activated dislocation motion made creep the dominant deformation mechanism, causing a continuous strain increase. However, as stress relaxation was completed, the strain rate gradually approached zero, and strain growth stagnated. During the low-temperature holding stage, atomic diffusion is suppressed, and dislocation motion is impeded, causing the material to exhibit near-elastic behavior. Concurrently, yield strength and elastic modulus significantly increase, enhancing resistance to deformation. This results in a viscous-plastic strain rate approaching zero and strain remaining stable. At this point, the magnitude of compressive creep is substantially lower than the tensile inelastic strain observed at high temperatures. It should be noted that during the initial heating phase, the instantaneous temperature change rate reaching 20 °C/min triggers an exceptionally high strain rate response. Consequently, the equivalent viscoplastic strain growth rate during this period is markedly higher than in other phases.

### 3.3. Fatigue Life Prediction and Discussion

The failure mechanism for solder joint thermal cycling primarily involves low-cycle fatigue. Therefore, the analysis is based on the Coffin–Manson equation modified by Engelmaier [55], expressed as follows:

(1) $$N_{f}=\frac{1}{2}{\left(\frac{{∆\gamma }_{\tau }}{2\varepsilon_{f}}\right)}^{\frac{1}{c}}$$

In the above formula:

N_f_ denotes fatigue life;

ε_f_ represents the fatigue toughness coefficient;

c is the fatigue toughness index;

Δγ_τ_ denotes the cyclic equivalent viscoplastic shear strain range of the welded joint, $\Delta \gamma_{\tau }\;=\sqrt{3}\Delta \varepsilon$;

c is a parameter related to the temperature cycle profile and can be determined by the following equation:

(2) $$∁\;=-0.442-0.0006T_{\mathrm{sj}}\;+\;0.0174\ln\left(1\;+\;f\right)$$

where

f is the cycle frequency (in cycles/day);

T_sj_ is the average cycle temperature of the temperature cycle (in °C).

Failure of micro-projection welds typically occurs when the total strain reaches its peak value. To evaluate its fatigue performance, it is necessary to analyze the equivalent viscoplastic strain range Δε and the cyclic equivalent plastic shear strain range Δγ_τ_. Data from the fourth cycle in Figure 8 indicate that the equivalent viscoplastic strain range for the critical weld is Δε = 0.00513. The equivalent plastic shear strain range is Δγ_τ_ = 0.0089.

Based on the average cyclic temperature T_sj_ of 35 °C and a daily experimental cycle frequency f = 24, the parameter value is calculated as ∁=−0.407, with ε_f_ set to 0.325 [56,57,58,59]. Consequently, the thermal fatigue life of this weld joint is determined to be 18,930 cycles.

**Figure 9:**
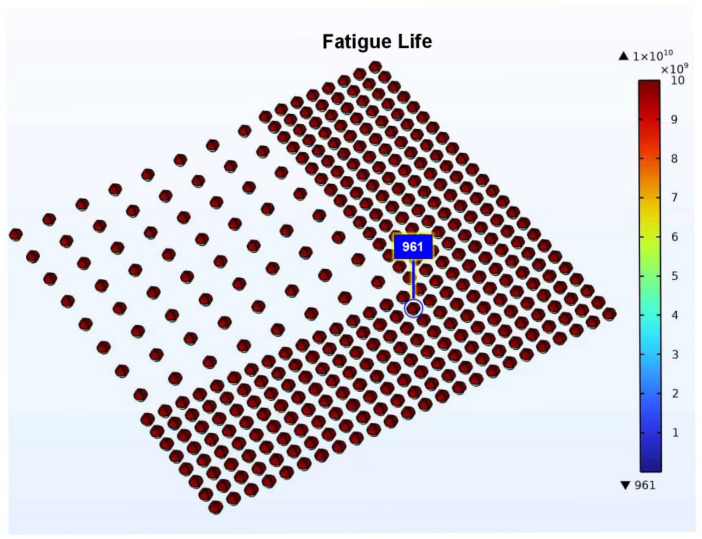
Fatigue life values from the fatigue module in COMSOL software.

The fatigue life of the weld joint calculated using the COMSOL Multiphysics Fatigue Module is 961 cycles (Figure 9), which differs by approximately 20 times from the predicted life value (18,930 cycles) based on strain data from the fourth cycle’s steady-state phase in this study. This discrepancy primarily stems from the differing strain ranges employed. The COMSOL module utilizes the transient response from the first temperature cycle, where the strain range peaks due to initial thermal shock effects. This range incorporates substantial non-repeatable elastic strain and initial plastic overshoot components, leading to a severe overestimation of single-cycle damage and consequently a significant underestimation of fatigue life. This significant discrepancy directly demonstrates the practical consequences of overlooking the mechanistic distinction between transient and stabilized responses. The fatigue damage in viscoplastic materials like Sn-based solder primarily accumulates through steady-state creep under typical packaging thermal cycles [60]. Therefore, our findings mandate that accurate life predictions be based on stabilized cyclic data. Consequently, reliability testing protocols and damage assessments must be anchored in steady-state material behavior to ensure a valid correlation between accelerated tests and in-service lifetime.

### 3.4. Sensitivity Analysis

The preceding analysis established a significant discrepancy between fatigue life predictions based on the initial transient cycle and those derived from the stabilized cyclic response. To ensure that this core finding accurately reflects the underlying physics rather than being an artifact of numerical choices or parameter uncertainty, a comprehensive set of verification and sensitivity analyses was conducted. This section presents the results of mesh convergence studies and parameter sensitivity assessments, which collectively validate the reliability of the simulation framework and the conclusions drawn from it.

To verify that the predicted fatigue life is not significantly affected by mesh discretization, a systematic mesh convergence study was conducted. Three mesh densities were examined for the sub-model: coarse (1749 elements), reference (5155 elements), and refined (9851 elements). The predicted fatigue life at key points is summarized in Table 6. As shown, when the mesh is refined from the reference to the refined level, the change in transient fatigue life is only 3.5%. More importantly, the predicted transient and steady-state lives remain stable across the two finer meshes, and their ratio consistently differs by a factor of approximately 20. This demonstrates that the numerical uncertainty introduced by mesh discretization is far smaller than the core physical phenomenon under investigation—namely, the order-of-magnitude difference between transient and steady-state failure. Therefore, the numerical results do not affect the comparison of failure stages or the conclusions drawn from them.

Having established the numerical reliability (mesh convergence analysis) of the results, we further evaluated their sensitivity to key modeling parameters. An analysis of the fatigue toughness coefficient (ε_f_) shows that varying it by ±20% around 0.325 substantially changes the absolute predicted lives, as expected from the Coffin–Manson model (Table 7). Crucially, the ratio between steady-state and transient lives remains stable at approximately 19.7–19.8. This demonstrates that the core finding—a steady-state life ~20 times greater than the transient life—is robust and insensitive to ε_f_ within its accepted range. Thus, the dominant factor driving the discrepancy is the methodological choice itself.

Subsequently, the sensitivity to the constitutive parameters of the solder material itself was examined. We quantitatively compare two key Anand parameters—the pre-exponential factor and the activation energy. Table 8 shows their effects on predicted transient life, steady-state life, and their ratio. The results show that the ratio of steady-state to transient life remains notably stable with variations in the pre-exponential factor, ranging only from 19.69 to 20.11. In contrast, changes in the activation energy cause a more pronounced fluctuation in this ratio, which varies from 18.04 to 21.15. Despite this wider range for Q/R, both parameters maintain a consistent order-of-magnitude difference between the two life stages. This shows that the order-of-magnitude difference between assessment methods is not due to Anand parameter choices; rather, it is a consistent feature of the modeled thermo-mechanical response.

The robustness of the simulation framework was fully verified through systematic sensitivity analyses of mesh independence, Anand constitutive parameters, and fatigue toughness coefficients. This provides confidence for further elucidation of the underlying physical mechanisms and methodological rationale. Specifically, thermal fatigue in solder joints results from the cyclic accumulation of viscoplastic strain, driven primarily by creep mechanisms during stabilized cycling. The initial transient cycle does not govern the damage process; instead, it is the progressive accumulation of viscoplastic strain over stabilized cycles that determines fatigue life. Therefore, to accurately predict fatigue life using the Coffin–Manson model, the equivalent viscoplastic strain range must be extracted after the cyclic response has stabilized. This approach directly captures the incremental nature of plastic strain accumulation under repeated loading. To further improve the applicability of this methodology, future research will extend the investigation to a broader range of solder alloy systems. This will involve experimental calibration of the full set of material parameters for different alloys, with the goal of establishing a generalized framework for the design of high-reliability solder joints.

## 4. Conclusions

This study proposes a hypothesis and a corresponding method for evaluating the thermal fatigue of solder joints, suggesting that analysis based on the cyclic steady-state response may provide a more physically grounded life assessment than traditional transient-cycle approaches. It demonstrates that the alternating thermal stress caused by material mismatch induces periodic shear strain, leading to strain localization and stress concentration at the solder–pad interface edge—the primary site for crack initiation and propagation. By introducing the “cyclic steady state” as a new benchmark and utilizing the viscoplastic strain energy density from the fourth stable cycle (ΔWvp = 0.00599 MJ/m^3^) as the damage parameter, a fatigue life of 18,930 cycles is predicted. This approach more accurately captures creep-dominated cumulative damage compared to first-cycle transient analysis. From an energy perspective, the high viscoplastic strain energy density at critical locations fundamentally drives crack evolution. By integrating strain distribution and energy dissipation, a quantitative reliability criterion is established, validated by the strong spatial correlation between high-ΔWvp regions and actual crack sites observed via SEM. These findings provide a key basis for developing a high-reliability life evaluation methodology for solder joints.

Collectively, this work offers systematic advances on three interconnected fronts. Mechanistically, it shifts the focus from traditional stress–strain analysis to an energy-based framework, establishing ΔWvp as the essential driver of crack growth. Methodologically, it defines and applies the “cyclic steady state” as a robust benchmark for life prediction, moving beyond the over-conservative estimates from first-cycle analysis. Evaluatively, it integrates the understanding of shear–strain localization, cyclic stabilization criteria, and energy dissipation metrics to formulate a comprehensive, quantitatively supported reliability assessment framework. Together, these contributions strengthen the theoretical foundation and provide practical guidance for the design of fatigue-resistant solder joints in high-reliability electronic packaging.

## Figures and Tables

**Table 1:** Model geometric parameters.

Structure	Diameter/μm	Spacing/μm	Height/μm	Quantity
Solder joint	40	80	40	384
Central copper pad	40 × 40	80	5	361
Surrounding copper pads	40 × 100	120	5	23
Silicon substrate	600 × 600	——	200	1

**Table 2:** Anand model parameters for Sn3.5Ag solder.

Parameter	Sn3.5Ag	Definition in Anand’s Model	Physical Significance
A (s^−1^)	22,300	Pre-exponential factor	Scales the overall inelastic strain rate.
Q/R (K)	8900	Activation energy	Determines the temperature sensitivity of the deformation process.
S_0_ (MPa)	39.09	Initial value deformation resistance	The initial value of the internal state variable representing slip resistance.
ξ	6	Multiplier on stress	Scales the effective stress to drive inelastic strain rate.
m	0.182	Strain rate sensitivity exponent for stress	Controls the strain rate dependence of the flow stress.
n	0.018	Strain rate sensitivity exponent for the saturation stress	Controls the strain rate dependence of the saturation value.
a	1.82	Strain rate sensitivity of hardening/softening	Controls the rate of evolution of deformation resistance.
S (MPa)	73.81	Saturation coefficient of deformation resistance	Amplitude of steady-state deformation resistance.
$h_{0}$ (MPa)	3321.15	Hardening constant	Governs the initial rate of change of deformation resistance.

**Table 3:** Material parameters of the modeling structure.

Material Properties	Cu	Si
Density $\left(kg/m^{3}\right)$	8960	2329
Coefficient of thermal expansion $\left(10^{-6}K^{-1}\right)$	17	2.6
Young’s modulus $\left(GPa\right)$	110	170
Poisson ratio	0.35	0.28
Thermal conductivity $[W/\left(m\;\times \;K\right)]$	400	130

**Table 4:** Material parameters of Sn3.5Ag.

Temperature (°C)	Young’s Modulus $\left(GPa\right)$	Coefficient of Thermal Expansion $\left(10^{-5}K^{-1}\right)$	Poisson Ratio
−55	59.9	1.66	0.349
−35	58.5	2.22	0.352
−15	57.1	2.66	0.354
5	55.5	2.96	0.357
25	54	3.16	0.36
45	52.3	3.27	0.363
65	50.6	3.32	0.367
85	48.9	3.33	0.371
105	47	3.32	0.374
125	45.1	3.31	0.378

**Table 5:** Parameters of the cycle period.

Cycle Period	Loop Area	Δε	ΔWvp
First	0.0056	0.023	0.0056
Second	0.00593	0.00531	0.00593
Third	0.00595	0.005	0.00595
Fourth	0.00597	0.00513	0.00597

**Table 6:** Mesh convergence analysis.

Mesh	Transient Life	Steady-State Life	Ratio
Coarse mesh	617	12,408	20.11
Reference mesh	961	18,930	19.69
Refined mesh	1014	19,622	19.35

**Table 7:** Sensitivity analysis results of ε_f_ parameters.

ε_f_	Transient Life	Steady-State Life	Ratio
0.26	555	10,997	19.814
0.325	961	18,930	19.698
0.39	1504	29,777	19.798

**Table 8:** Sensitivity analysis of the Anand key constants of Sn3.5Ag.

Parameters	Sn3.5Ag	Transient Life	Steady-State Life	Ratio
A (s^−1^)	18,955	969	19,491	20.11
	22,300	961	18,930	19.69
	25,645	949	18,847	19.86
Q/R (K)	7565	716	15,142	21.15
	8900	961	18,930	19.69
	10,235	1242	22,405	18.04

## Data Availability

The original contributions presented in this study are included in the article. Further inquiries can be directed to the corresponding authors.
